# Awareness of Hypoglycemic Episodes Among Patients With Type 2 Diabetes Mellitus in Aseer Region, Saudi Arabia: A Cross-Sectional Study in 2024

**DOI:** 10.7759/cureus.72374

**Published:** 2024-10-25

**Authors:** Fatima Alqahtani, Amjad BinGhamiah, Abrar Alqahtani, Anas Alqahtani, Yara A Alorfi, Rawan Alqahtani, Mohrah Alqahtani, Ali A Alshahrani

**Affiliations:** 1 Family Medicine, Armed Forces Hospital Southern Region, Aseer, SAU; 2 Medicine and Surgery, University of Jeddah, Jeddah, SAU; 3 Medicine and Surgery, College of Medicine, King Khalid University, Abha, SAU; 4 Specialist Nursing, Al-Buraidah Primary Health Care Center, Ministry of Health, Aseer, SAU; 5 Endocrinology and Metabolism, King Abdulaziz Medical City, Riyadh, SAU

**Keywords:** aseer, diabetes, hypoglycemia, hypoglycemic attacks, saudi arabia

## Abstract

Background

Hypoglycemia, where blood glucose is ≤70 mg/dL, is a serious diabetic complication with high individual and community costs. This study aimed to investigate the level of awareness of hypoglycemic episodes among type 2 diabetic patients in the Aseer region, Saudi Arabia.

Methods

This cross-sectional study was conducted in the Aseer region of Saudi Arabia over 9 months, among 235 type 2 diabetic patients. Data were collected via a pretested self-administered questionnaire and analyzed using SPSS v.25 (IBM Corp., Armonk, NY, US). The questionnaire included questions about socio-demographic factors and awareness of hypoglycemia among the adult population in the Aseer Region.

Results

A total of 235 type 2 diabetic patients were enrolled in this study; females formed the majority (60.4%). Regarding educational level, 27.2% of participants were uneducated, 26.4% had a high school education, and 11.1% had a university education. A history of hypoglycemic attack was positive in 50.6% of cases. Overall, about 12.9% of participants were found to have good awareness regarding episodes of hypoglycemia, compared to the majority (87.1%) who had a poor level of understanding. The most known causes and risk factors for the episodes of hypoglycemia were skipping meals or fasting (63.7%) and the use of hypoglycemic drugs (39.5%). More than half (55.3%) considered episodes of hypoglycemia as a life-threatening event, and a slightly higher percentage (55.7%) thought that it could lead to severe complications. Correlation testing showed a statistically significant association between gender, age, and occupation of participants and their level of awareness regarding hypoglycemia (P< .05).

Conclusions

The study concluded that the majority of patients had a poor level of awareness. A statistically significant association was observed between the gender, age, and occupation of participants and their level of awareness of hypoglycemic attacks.

## Introduction

Diabetes mellitus is defined as raised blood glucose levels due to several factors such as a defect in insulin secretion, increased insulin resistance at the receptors, or a combination of both. This condition is a major and widespread chronic metabolic disorder. It is categorized into different types: type I diabetes, which is attributed to severely decreased insulin secretion, and type 2 diabetes, which is characterized by insulin resistance and deficient insulin secretion [1,2].

Diabetes mellitus is globally prevalent, with projections indicating its prevalence may reach up to 700 million patients by 2045 [3]. In the majority of high-income nations, diabetes is the fourth leading cause of death. This condition can also affect multiple organ systems, leading to cardiovascular, neurological, renal, and ophthalmic complications, which result in severe morbidity and substantial medical costs. Additionally, acute complications of diabetes could include diabetic ketoacidosis and hyperosmolar hyperglycemic nonketotic coma [3].

Hypoglycemia, which is defined as blood sugar levels less than 70 mg/dl or less, is a notable complication of diabetes imposing significant financial burdens on both patients and healthcare systems [4]. Insulin-treated individuals are at increased risk of experiencing hypoglycemic episodes. Data indicate that insulin-associated hypoglycemia is primarily observed in patients with type I diabetes undergoing insulin therapy, resulting in approximately 98,000 emergency department visits and around 30,000 hospitalizations annually in the United States [5].

Evidence shows that hypoglycemia necessitating emergency medical care is prevalent in both types of diabetes [6]. A report from Saudi Arabia revealed a high rate of acute complications due to hypoglycemic episodes in 68% of individuals suffering from diabetes mellitus [7]. This finding is consistent with another Saudi study that found that impaired hypoglycemia awareness contributed to a high occurrence of hypoglycemic episodes in type I diabetes in (62%) of patients [8].

In response to low blood sugar, a counterregulatory mechanism is activated to address low blood sugar levels. This mechanism often presents with autonomic features such as palpitations, tremors, hunger, and sweating, which act as indicators of hypoglycemia awareness. If this low blood glucose is not promptly managed, neuroglycopenic symptoms may emerge, including dizziness and cognitive dysfunction, potentially progressing to seizures, coma, or death [9,10]. Life-threatening complications, such as cardiac arrhythmias, cognitive impairment, and cerebral ischemia, may occur [11]. Furthermore, repeated hypoglycemic episodes can diminish counterregulatory hormonal and sympathetic responses, leading to reduced awareness and an elevated risk of recurrent episodes of severe hypoglycemia [11].

Hypoglycemia imposes a considerable strain on healthcare systems, resulting in increased costs and productivity losses, which can compromise the quality of care [12]. Literature indicates that the likelihood of hypoglycemia is elevated in patients who have been using insulin for an extended period and have had a prolonged history of diabetes [13,14]. Additionally, severe hypoglycemic episodes are more prevalent among individuals with diminished awareness of hypoglycemia [15]. 

According to Kedia et al., the primary risk factors for severe hypoglycemia include inappropriate dietary practices (47%), intense exercise (23%), dose miscalculation (16%), and low hypoglycemia awareness (5%) [16]. Further reports have shown that individuals with low hypoglycemia awareness are prone to experiencing more severe hypoglycemic events [17]. In addition, though rare, in certain cases, it was reported that tight glycemic control of the non-diabetic range may increase the risk of hypoglycemia [18].

Another study conducted in India with 366 patients with type 2 diabetes found that around 34% had inadequate knowledge of hypoglycemia [19]. This insufficient awareness was correlated with advanced age, lack of literacy, and lower socioeconomic status [19]. A survey by the American Association of Clinical Endocrinology involving 2530 participants with type 2 diabetes stated that most of the participants lacked awareness about the risk factors and conditions associated with hypoglycemia [20]. Another survey conducted in the Najran region revealed that 44% of patients with diabetes had a limited understanding of the symptoms and signs of hypoglycemia [21].

Diabetes has become a major global health concern, and recent research indicates that increasing awareness among diabetic patients can improve glycemic control. Greater awareness enables patients to comprehend the risks associated with diabetes, encourages them to pursue adequate medical management, and provides education on effective disease management and control [22]. This study aims to examine and enhance the awareness and understanding of hypoglycemic episodes among type 2 diabetic individuals in the Aseer region of Saudi Arabia. The research seeks to address a significant gap in existing medical literature and aligns with the national objective of advancing patient care and improving clinical outcomes across the kingdom.

## Materials and methods

Study design

In this cross-sectional study, we explored Aseer City over a month, from August to September 2024, to examine awareness, risk factors, and factors associated with the level of awareness toward hypoglycemic episodes among type 2 diabetic patients.

Study population

The study population consisted of 235 type 2 diabetic participants from Aseer, Saudi Arabia, diagnosed with diabetes. We employed convenience sampling, allowing participants to join based on their willingness and consent. Initially, 235 individuals were considered. 

Inclusion criteria

The inclusion criteria encompassed diabetic patients (18 to 60 years old) residing in Aseer, Saudi Arabia. Informed consent was provided by participants. 

Exclusion criteria

The exclusion criteria comprised diabetic patients outside the specified age range, those residing outside Aseer, Saudi Arabia, and those with medical conditions or comorbidities such as hypertension and heart issues. Additionally, patients not willing to give consent were excluded.

Data collection

Data were collected through interviews with patients attending the Family Medicine clinics at King Fahad Armed Forces Hospital in Aseer, Saudi Arabia. The questionnaire was validated [23] and contained inquiries regarding socio-demographic factors and the awareness of hypoglycemic episodes among adults. This comprehensive approach aimed to ensure thorough and inclusive data collection related to the awareness of hypoglycemic attacks.

Data analysis

Data were initially entered and cleaned using Microsoft Excel (Microsoft Corporation, Redmond, WA, US) and then analyzed using SPSS version 25 (IBM Corp., Armonk, NY, US). Data were presented as numbers and percentages and displayed in the form of tables and figures. For inferential statistics, a P-value of <0.05 was considered significant.

Ethical considerations

Formal approval from the Research Ethics Committee at the King Fahad Armed Forces Hospital was received via Request Code: AFHSRMREC/2024/Family Medicine/740 on August 18, 2024. Informed consent was obtained from all participants.

## Results

This study included 235 type 2 diabetic patients from the Aseer Region; females represented 60.4% while males constituted the remaining (39.6%). Almost all participants were Saudis (99.6%). The most common age group was 58 years and more (50.6%). Regarding educational level, 27.2% of participants were uneducated, 26.4% had a high school education, and 11.1% had a university education. The overwhelming majority (87.2%) were married individuals. Over half (56.1%) were housewives while 86.8% were residents of urban areas. Fifty-two point three percent (52.3%) had a monthly income of less than 3000 SAR (about 800 USD) (Table 1).

**Table 1:** Sociodemographic characteristics of study participants (N=235) Descriptive statistics presented as frequencies and percentages

Characteristics	Subgroups	Frequency	Percent
Gender	Male	93	39.6
	Female	142	60.4
Nationality	Saudi	234	99.6
	Not Saudi	1	.4
Age (years)	18-28 years	3	1.3
	29-38 years	5	2.1
	39-48 years	33	14.0
	49-58 years	75	31.9
	≥ 58 years	119	50.6
Education level	Uneducated	64	27.2
	Primary school	42	17.9
	Intermediate	41	17.4
	High school	62	26.4
	University and above	26	11.1
Marital status	Single	4	1.7
	Married	205	87.2
	Widowed	26	11.1
Status of employment	Part-time	3	1.3
	Full time	24	10.8
	Housewife	125	56.1
	Retiree	71	31.8
Residence	Urban	204	86.8
	Rural	31	13.2
Average monthly income (SAR)	< 3000	123	52.3
	3000 - 4999	31	13.2
	5000 - 9999	41	17.4
	10000 - 14000	31	13.2
	15000 or more	9	3.8

Analysis revealed that 99.6% of patients in the study were taking medications for the management of diabetes. Twenty-one point seven percent (21.7%) were taking insulin, and (63%) were taking oral hypoglycemic medications. The majority of participants (85.5%) reported being familiar with the term hypoglycemia or hypoglycemic episodes. Regarding the information provided for patients about hypoglycemic episodes, 53.2% agreed that it is sufficient (Table 2).

**Table 2:** Used medications for diabetes management and had awareness about hypoglycemic episodes (N=235) Descriptive statistics are presented as frequencies and percentages.

Statement	Answer	Frequency	Percent
Are you taking any medications for diabetes management?	Yes	234	99.6
	No	1	.4
Are you taking insulin?	Yes	51	21.7
	No	184	78.3
Are you taking oral hypoglycemic medications?	Yes	148	63.0
	No	87	37.0
Are you familiar with the term hypoglycemia or hypoglycemic episodes?	Yes	201	85.5
	No	34	14.5
Do you think diabetic patients are given sufficient information about hypoglycemic episodes?	Yes	125	53.2
	No	110	46.8

**Figure 1:**
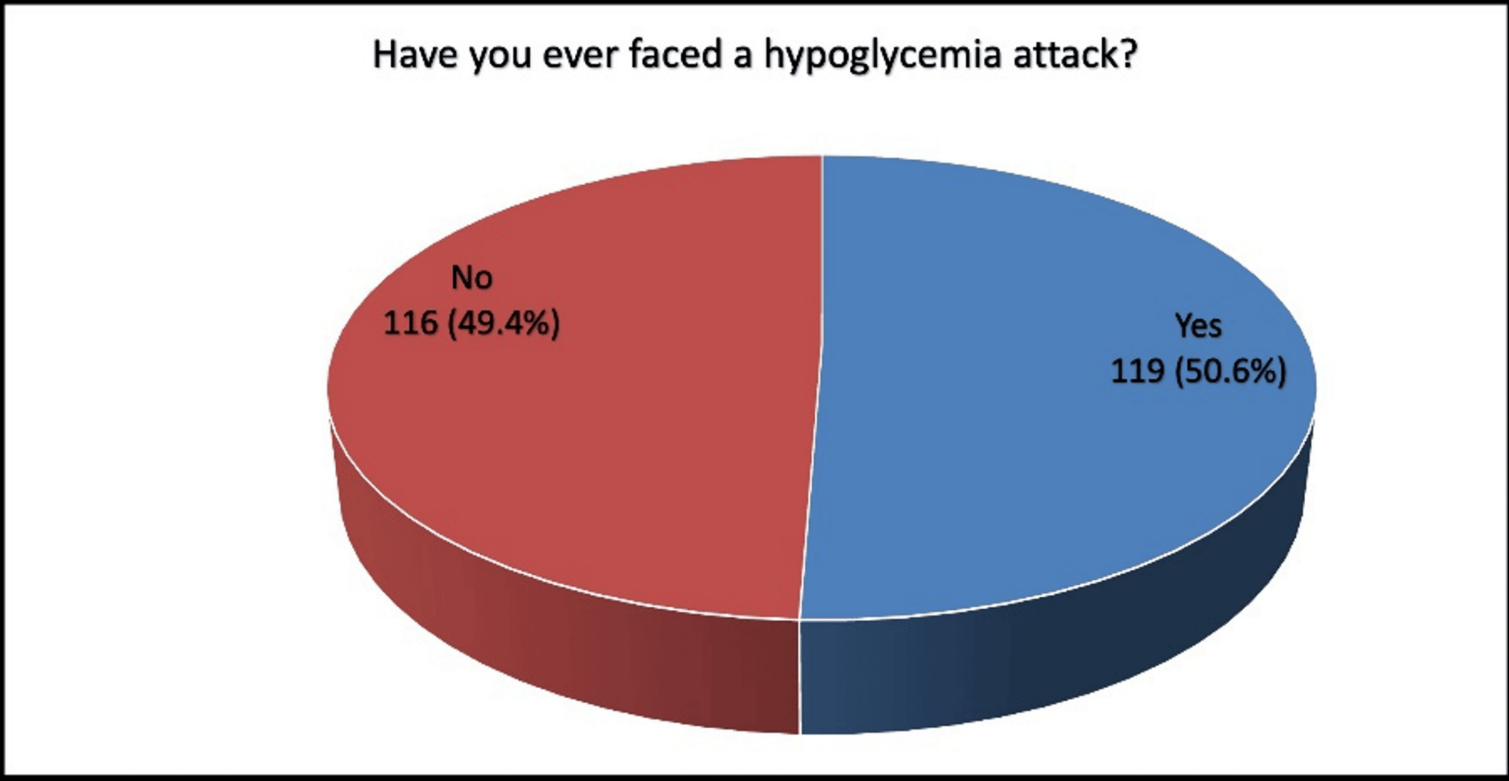
History of episodes of hypoglycemia, (N=235)

History of hypoglycemic episodes was positive in 50.6% of cases (Figure 1). 

**Figure 2:**
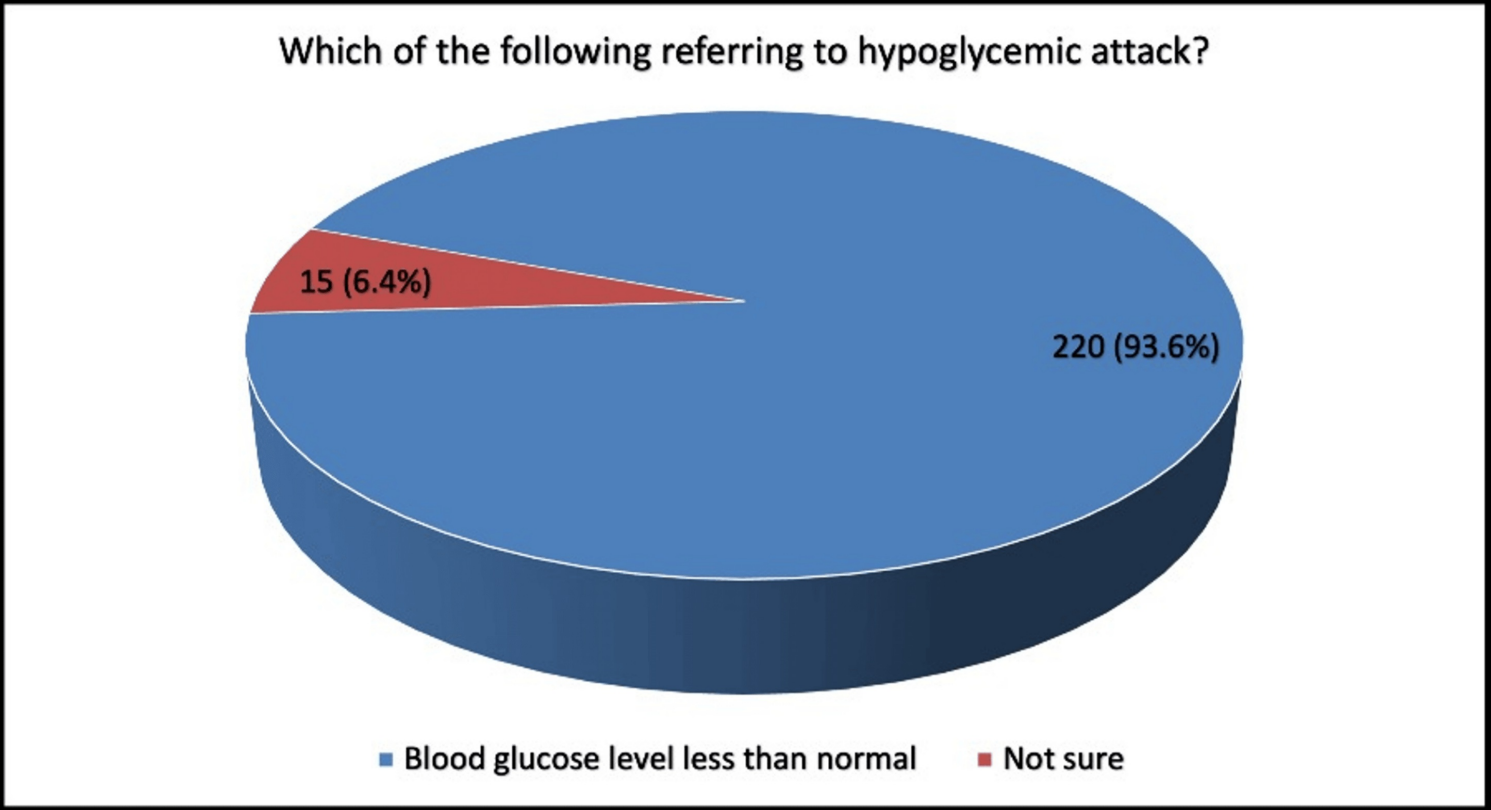
Definition of episodes of hypoglycemia, (N=235)

The majority of participants (93.6%) defined episodes of hypoglycemia as a blood glucose level less than normal while 6.4% were uncertain (Figure 2).

**Figure 3:**
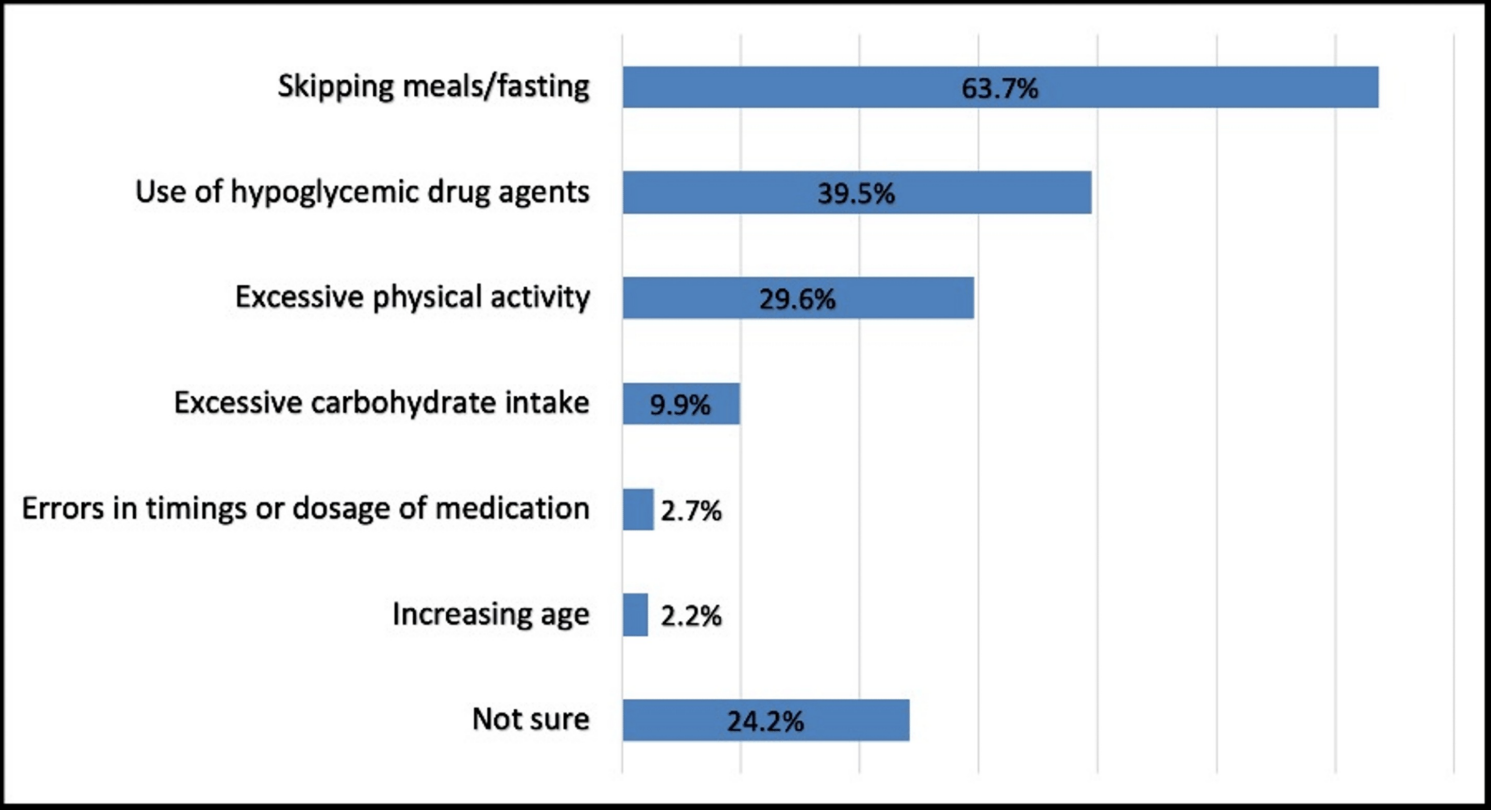
Common causes or risk factors for hypoglycemic attacks

The most known causes and risk factors for hypoglycemic episodes were skipping meals or fasting (63.7%) and the use of hypoglycemic drugs (39.5%) (Figure 3).

Known treatment strategies included eating any food, especially sweets (74.4%) and drinking fruit juice (60.5%). Regarding prevention methods, 24.8% agreed on regular and frequent glucose level monitoring (Table 3).

**Table 3:** Participants’ awareness regarding hypoglycemic episodes, (N=235) Descriptive statistics presented as frequencies and percentages

Statement	Answer	Frequency	Percent
What are the treatment strategies for a hypoglycemia event?	Eating any food, especially something sweet	166	74.4%
	Drinking fruit juice	135	60.5%
	Taking 15-20 g glucose tablets	3	1.3%
	None of these	3	1.3%
	Not sure	33	14.8%
How one can prevent hypoglycemic episodes?	Eating sugar-rich food daily	6	2.7%
	Maintaining a routine of healthy food	43	19.4%
	Work	4	1.8%
	Exercise and sleep	41	18.5%
	Regular and frequent glucose level monitoring	55	24.8%
	Reporting low blood glucose level episodes to the physician for dosage adjustment	13	5.9%
	Strictly following medication and diet guidelines recommended by the physician	32	14.4%
	Not sure	84	37.8%
Do you think hypoglycemic episodes can be life-threatening?	Yes	130	55.3
	No	5	2.1
	Not sure	100	42.6
Do you think hypoglycemic events can lead to severe complications?	Yes	131	55.7
	No	4	1.7
	Not sure	100	42.6

**Figure 4:**
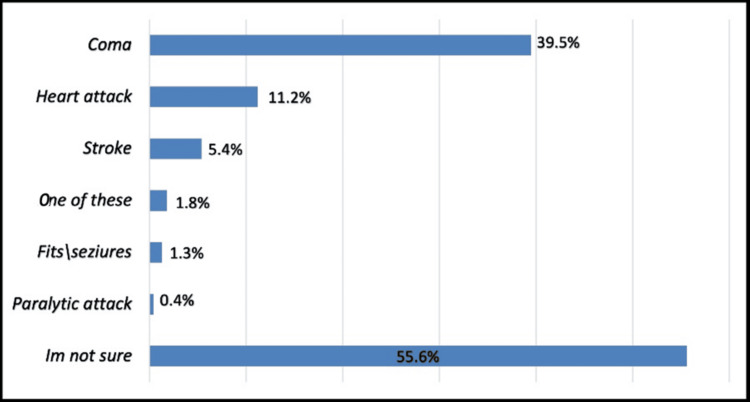
Possible complications of frequent hypoglycemic episodes in diabetic patients

More than half (55.3%) considered hypoglycemic episodes as a life-threatening event, a slightly higher percent (55.7%) thought that it could lead to severe complications, the majority (55.6%) were not sure about these complications while 39.5% correctly identified coma. Overall, 12.9% of participants were found to have good awareness of hypoglycemic episodes while 87.1% had a poor level of awareness (Figure 4).

Correlation testing revealed a significant association between gender, age, and occupation of participants with their level of awareness regarding hypoglycemic episodes (P < .05) (Table 4).

**Table 4:** Factors associated with level of awareness regarding hypoglycemic episodes, (N=235) Significant association with a p-value < 0.05 (chi-square test)

Factors	Subgroups	Level of awareness		
		Poor	Good	P value
Gender	Male	78.3%	21.7%	.02*
	Female	92.3%	7.7%	
Age (years)	18-28 years	100.0%	0.0%	.006*
	29-38 years	0.0%	100.0%	
	39-48 years	78.6%	21.4%	
	49-58 years	82.9%	17.1%	
	≥ 58 years	93.9%	6.1%	
Education level	Uneducated	94.9%	5.1%	.2
	Primary school	94.7%	5.3%	
	Intermediate	81.0%	19.0%	
	High school	78.8%	21.2%	
	University and above	83.3%	16.7%	
Marital status	Single	100.0%	0.0%	.7
	Married	85.8%	14.2%	
	Widowed	94.1%	5.9%	
Status of employment	Part time	0.0%	100.0%	.005*
	Full time	63.6%	36.4%	
	Housewife	93.2%	6.8%	
	Retired	84.6%	15.4%	
Residence	Urban	87.7%	12.3%	.7
	Rural	83.3%	16.7%	
Average monthly income (SAR)	< 3000	92.5%	7.5%	.06
	3000 - 4999	88.2%	11.8%	
	5000 - 9999	66.7%	33.3%	
	10000 - 14000	87.5%	12.5%	
	15000 or more	100.0%	0.0%	
Significant association with a p-value < .05				

## Discussion

Routine practice has shown that achieving optimal blood sugar control can be challenging, as it requires careful management of glycemic levels while also considering the risk of hypoglycemia. Balancing these factors is crucial for effective diabetes management [24,25]. Patients must be educated and made aware of the signs of hypoglycemia so they can identify them early and take the necessary actions to address the situation. Early recognition and intervention are key to preventing severe complications associated with hypoglycemia. This study aims to assess the level of awareness of hypoglycemic episodes among diabetic patients in the Aseer region of Saudi Arabia. A total of 235 diabetic patients were enrolled in this study, of which, females represented the majority (60.4%). Regarding educational level, 27.2% of participants were uneducated, 26.4% had a high school education, and 11.1% had a university education. It is important to address the educational levels of the participants to get prior insight into the expected level of awareness.

Analysis revealed that 99.6% of patients in the study were taking medications for the management of diabetes. (21.7%) were taking insulin, and (63%) were taking oral hypoglycemic medication. Literature reported that patients on insulin are more likely to suffer from severe hypoglycemia, most probably due to insulin dose miscalculation [14,15,17]. The majority of participants (85.5%) reported being familiar with the term hypoglycemia or hypoglycemic episodes. History of hypoglycemia was positive in (50.6%) of cases. A previous Saudi study included 400 patients with DM. The study reported that the incidence of hypoglycemia among the total studied diabetic patients was 90 (22.5%) in a year [26]. Another Saudi research revealed that more than two-thirds of the participants (72.1%) reported experiencing hypoglycemic episodes within the past week [27]. These high percentages highlight the need for enhanced treatment protocols and more effective patient education strategies to ensure optimal management.

The majority of participants (93.6%) defined hypoglycemia as a blood glucose level less than normal. Overall, about 12.9% of participants were found to have a good awareness regarding hypoglycemia, compared to the majority (87.1%) who had a poor level of awareness. This contrasts with a similar study conducted in Saudi Arabia, which found that 61.4% of patients had a good level of knowledge regarding hypoglycemia and only 38.6% had a poor level of knowledge. This indicates a higher awareness and understanding of hypoglycemia among Saudi Arabian patients compared to the findings in other studies. Another Indian study reported that 66.1% of diabetic patients had good knowledge of hypoglycemia [19]. This poor level of knowledge is an alarming sign and a predictor of potential episodes of hypoglycemia.

The most known causes and risk factors for hypoglycemic episodes were skipping meals or fasting (63.7%) and the use of hypoglycemic drugs (39.5%). This is compatible with a previous Saudi study where it was found that the most common risk factor associated with hypoglycemia was eating after a long period of insulin intake, and the most common symptoms of hypoglycemia were tremors, sweating, palpitations, and drowsiness [26].

Known treatment strategies included eating any food, especially sweets (74.4%) and drinking fruit juice (60.5%). These findings are in agreement with a previous study in Nepal [28], where about 49% of diabetic patients preferred taking glucose powder or sugar with water as an immediate measure to treat hypoglycemia. Regarding prevention methods, 24.8% agreed on regular and frequent glucose level monitoring. These findings are compatible with previous studies showing similar results with most participants reporting increased calorie intake to prevent hypoglycemia [29].

More than half (55.3%) considered hypoglycemia as a life-threatening event, and a slightly higher percentage (55.7%) thought that it could lead to severe complications. In Saudi Arabia, it was found that 68.9% of patients with diabetes experienced a high rate of acute complications, where 65.4% experienced ketoacidosis and 68.9% experienced hypoglycemic attacks [7].

Correlation testing revealed a statistically significant relationship between participants' gender, age, and occupation and their awareness level concerning hypoglycemia (P < .05). This is almost compatible with a study in South India, where it was observed that poor awareness level was correlated to older age, illiteracy, and low socioeconomic status [19]. Therefore, efforts to enhance the patient's socioeconomic status should be arranged to secure suitable financial support, particularly for the elderly and the underprivileged.

To the best of our knowledge, there have been few Saudi studies published on this topic, making this research a valuable foundation of evidence. Another strength of the study is its inclusion of participants from diverse demographic and socio-economic backgrounds, which can help authorities address the issue from multiple perspectives. Additionally, this study lays the groundwork for future research in this area. However, it is not without limitations. Since the project was conducted in a single region, the sample may represent a very particular group of cases, making it challenging to generalize the findings to the entire diabetic population in the kingdom.

## Conclusions

The study concluded that the majority of patients had a poor level of awareness. The most known causes and risk factors for hypoglycemic episodes were skipping meals or fasting and the use of hypoglycemic drugs. The study also revealed a statistically significant relationship between the participants' gender, age, and occupation and their level of awareness about hypoglycemic episodes (P< 0.05). Ongoing and frequent studies on this issue should be conducted and adequately funded to produce more evidence and data. Medical initiatives, educational campaigns, and workshops should also be organized to discuss and implement strategies focused on reducing the incidence of hypoglycemia.
